# Severe methanol poisoning treated with a novel hemodialysis system: a case report, analysis, and review

**DOI:** 10.1186/s41100-021-00362-8

**Published:** 2021-08-03

**Authors:** Alisa C. Illescas, Christos P. Argyropoulos, Sara A. Combs, Saeed K. Shaffi, Zhi Q. Xu, Michael A. Aragon, J. Pedro Teixeira

**Affiliations:** 1 Department of Internal Medicine, University of New Mexico School of Medicine, MSC10-5550, 1 University of New Mexico, Albuquerque, NM 87131 USA; 2 Division of Nephrology, University of New Mexico School of Medicine, MSC10-5550, 1 University of New Mexico, Albuquerque, NM 87131 USA; 3 Outset Medical, 3052 Orchard Drive, San Jose, CA 95134 USA; 4 Division of Pulmonary, Critical Care, & Sleep Medicine, University of New Mexico School of Medicine, MSC10-5550, 1 University of New Mexico, Albuquerque, NM 87131 USA

**Keywords:** Methanol poisoning, Toxic alcohol, Hemodialysis, Hemodialysis delivery systems, Kinetics

## Abstract

In May and June 2020, an outbreak of methanol poisoning arose in the southwest United States linked to ingestion of contaminated hand sanitizer imported during the coronavirus disease 2019 pandemic, ultimately resulting in over a dozen hospitalizations and at least four deaths in New Mexico and Arizona. In this report, we describe one of these cases in which profound methanol intoxication was successfully treated with the Tablo® Hemodialysis System, the first reported case of toxic alcohol poisoning treated with this novel device. We carry out a formal regression analysis of the serial methanol levels obtained in this case to conservatively estimate that intermittent hemodialysis with Tablo achieved a clearance of methanol of 239 mL/min (95% confidence interval, 173–305 mL/min), a clearance that is well within the previously published standard of care. We conclude by reviewing both the treatment of toxic alcohol poisoning and the determinants of small molecule clearance with hemodialysis, emphasizing the importance of optimizing the dialytic treatment of intoxications with extended treatment times and the use of high-efficiency dialyzers.

## Background

Toxic alcohol poisoning continues, even in the twenty-first century, to account for dozens of deaths annually in the United States (US) [1, 2]. Worldwide, it has been estimated that hundreds of people have died in 2020 alone related to misuse of methanol during the coronavirus disease 2019 (COVID-19) pandemic [3]. Early diagnosis and treatment of toxic alcohol poisoning remain essential to preventing morbidity and mortality. In this report, we present and analyze a case of severe methanol intoxication that was successfully treated with intermittent hemodialysis (IHD) using the Tablo® Hemodialysis System (Outset Medical, San Jose, CA, USA), the first reported case of toxic alcohol poisoning treated with this novel device.

## Case presentation

A 36-year-old man with alcohol dependence was brought by emergency medical services (EMS) to a local emergency department (ED) after being found down by his family with depressed mental status and slurred speech. He was initially diaphoretic and hypotensive with pupils fixed bilaterally at 5 mm. EMS administered a 2-L saline bolus with normalization of his blood pressure. However, due to his mental status (with a reported Glasgow Coma Scale of 6–7), endotracheal intubation was performed for airway protection soon after arrival to the ED. Initial labs were reportedly notable for an undetectable blood ethanol level and an anion gap of > 40 mmol/L. He was given fomepizole and transferred to our facility.

**Fig. 1:**
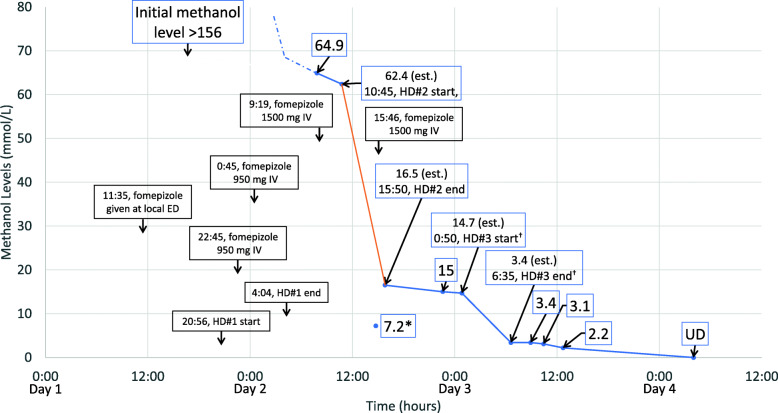
Methanol levels (measured and estimated) and treatment events. Included are measured methanol levels as well as estimated levels at the start and end of hemodialysis treatments #2 and #3. The drop in methanol levels during the Tablo treatment (HD#2) is highlighted in orange. The estimated methanol levels at the start and end of HD#2 and HD#3 were calculated assuming an endogenous methanol half-life (in the presence of ADH inhibition) of 52 h. ADH, alcohol dehydrogenase; ED, emergency department; HD, hemodialysis; UD, undetectable. To convert methanol levels from mmol/L to mg/dL, multiply by 3.2. *This level of 7.2 mmol/L (23 mg/dL) was drawn off of the dialysis catheter during treatment and is felt to be spurious. ^†^Note that there was an 85-min interruption in HD#3 which is not graphically depicted

Upon arrival later that afternoon to our medical intensive care unit, his exam was notable for stable vital signs but persistently depressed mental status with fixed symmetric pupils. He weighed 100 kg. Admission labs (see Table 1) were notable for a persistent anion gap metabolic acidosis and an extremely elevated osmolar gap of > 200 mOsm/kg. Toxicology and nephrology were consulted. A toxic alcohol panel was drawn and additional IV fomepizole was administered with IV folate, thiamine, and pyridoxine. Vascular access for hemodialysis (HD) was placed in the right internal jugular vein (15-cm 12-French Power-Trialysis catheter, Bard Access Systems, Salt Lake City, UT). Computed tomography (CT) of the head revealed “symmetric hypoattenuation in the bilateral basal ganglia involving portion of the putamen and external capsule… suspicious for hypoxic-ischemic injury or potentially toxic-metabolic injury in the correct clinical setting.” The alcohol panel results obtained just prior to the initiation of HD revealed a methanol level above the upper limit of detection (> 156 mmol/L or > 500 mg/dL). The patient received three total HD sessions. His first treatment was interrupted due to poor function of his vascular access, which persisted despite the use of in-line heparin, ultimately requiring a second dialysis access to be placed (30-cm right femoral Power-Trialysis). The first two treatments were performed with the Tablo Hemodialysis System. The third treatment was performed with a conventional IHD machine (Gambro Phoenix, Baxter International, Deerfield, IL, USA) and was also interrupted due to circuit clotting. All dialysis treatments utilized a Revaclear™ dialyzer [Baxter International, polyarylethersulfone, membrane surface area 1.4 m^2^, *K*_*O*_*A* for urea 1170 mL/min at blood flow rate (*Qb*) of 300 mL/min and dialysate flow rate (*Qd*) of 500 mL/min, *K*_*UF*_ 50 mL/h/mmHg]. See Table 2 and Fig. 1 for treatment sequence and serial methanol levels.

**Table 1:** Labs upon admission to the medical ICU

Lab	Result	Units
WBC	21.2 (H)	10^9^/L
Hemoglobin	146	g/L
Hematocrit	0.49	
MCV	9.2	10^−14^ L/cell
Platelets	263	10^9^/L
INR	1.08	
Sodium	139	mmol/L
Potassium	5	mmol/L
Chloride	107	mmol/L
Total CO_2_	7 (L)	mmol/L
BUN	4.6	mmol/L
Creatinine	133	μmol/L
Glucose	8.0	mmol/L
Calcium	1.6 (L)	mmol/L
Phosphorus	1.3	mmol/L
Magnesium	1.1	mmol/L
Anion gap	25 (H)	mmol/L
Total protein	77	g/L
Albumin	33 (L)	g/L
AST*	66 (H)	unit/L
ALT	43	unit/L
Alkaline phosphatase	136	unit/L
Total bilirubin	5.1	μmol/L
Ammonia*	34 (H)	μmol/L
Serum osmolality	530	mOsm/kg
Lactate	2.3	mmol/L
Lipase*	988 (H)	unit/L
Troponin I	< 0.017	μg/L
Creatine kinase	637 (H)	unit/L
Ethanol	ND	mmol/L
Acetaminophen	< 2	μmol/L
Salicylate	< 2	mmol/L
Ketones by urinalysis	Small	
Urine amphetamine screen	ND	
Urine barbiturate screen	ND	
Urine benzodiazepine screen	Positive	
Urine cannabinoid screen	Positive	
Urine cocaine screen	ND	
Urine methadone screen	ND	
Urine opiate screen	ND	
pH	7.23 (L)	
pCO_2_	18 (L)	mmHg
pO_2_	124	mmHg
Calculated HCO_3_	7 (L)	mmol/L
SaO_2_	98	%
Base deficit	19 (H)	mEq/L
FiO_2_	40	%

*ICU*, intensive care unit; *ND*, not detected. Abnormal numerical values are marked as high (H) or low (L). *Upper limit of normal for AST is 58 unit/L, for ammonia is 33 μmol/L, for creatine kinase is 242 unit/L, and for lipase is 360 unit/L

**Table 2:** Treatment events including hemodialysis prescription

Day	Time	Methanol levels (mmol/L)	Events
1	11:35		Fomepizole given at local ED.
1	16:40	> 156	
1	20:56		Treatment #1 started. Tablo® machine, Revaclear™ dialyzer, right IJ 15-cm 12-French Power-Trialysis™ catheter, lines reversed, *Qb* 350 mL/min, *Qd* 300 mL/min, UF 75 mL/h.
1	22:15		Circuit clotted.
1	22:44		Restarted with circuit lines changed and in-line heparin^†^ added. *Qb* 300 mL/min, *Qd* 300 mL/min.
1	22:45		Fomepizole 950 mg IV.
1	23:05		Due to poor flows, *Qb* dropped further to 250 mL/min.
1	23:14		Treatment stopped again. Unable to aspirate from either line. New vascular access placed (right femoral 30-cm Power-Trialysis catheter).
2	0:45		Fomepizole 950 mg IV.
2	0:56		Treatment restarted using femoral line. *Qb* 350 mL/min, *Qd* 300 mL/min, UF 200 mL/h.
2	4:04		Treatment #1 completed.
2	7:51	64.9	
2	9:19		Fomepizole 1500 mg IV.
2	10:45		Treatment #2 started. Tablo machine, Revaclear dialyzer, access 1-and-1 (right IJ arterial, right femoral venous return), *Qb* 350 mL/min, *Qd* 300 mL/min, UF 337 mL/h.
2	14:00		UF dropped to 117 mL/h.
2	14:45	7.2*	*Level measured from the dialysis catheter during HD.
	15:46		Fomepizole 1500 mg IV.
2	15:50		Treatment #2 completed.
2	22:36	15	
3	0:50		Treatment #3 started. Gambro Phoenix™ machine, Revaclear dialyzer, *Qb* 350 mL/min, *Qd* 700 mL/min, UF 100 mL/h.
3	4:10		Circuit clotted; treatment stopped.
3	5:35		Treatment #3 restarted. *Qb* 350 mL/min, *Qd* 700 mL/min, UF 200 mL/h.
3	6:35		Treatment #3 completed.
3	8:54	3.4	
3	10:25	3.1	
3	12:42	2.2	
4	4:00	ND	

*ED*, emergency department; *HD*, hemodialysis; *IJ*, internal jugular; *K*_*O*_*A*, product of the mass transfer coefficient and dialyzer membrane surface area; *ND*, not detected; *Qb*, blood flow rate; *Qd*, dialysate flow rate; *UF*, ultrafiltration rate. To convert methanol levels from mmol/L to mg/dL, multiply by 3.2. ^†^Note that, though used in this case, the most recent EXTRIP guideline recommends avoiding systemic anticoagulation with hemodialysis due to reports of intracranial hemorrhage occurring in a substantial minority of patients with methanol poisoning [4, 23]

Following the three sessions of HD, his anion gap, osmolar gap, and blood gas normalized. His methanol levels decreased and became undetectable on hospital day 4. He was extubated on hospital day 3 but had significant agitation and alcohol withdrawal leading to reintubation the next day. On hospital day 6, repeat head CT showed “further progression of vasogenic edema within the region of the basal ganglia bilaterally when compared to prior CT… consistent with known diagnosis of [methanol] ingestion.” By hospital day 8, his mental status improved sufficiently to allow for extubation. However, despite overall clinical improvement, the patient had significant visual impairment. He was examined by ophthalmology and had severe limitation of his visual acuity (e.g., finger-counting limited to 8–12 inches). The patient reported that the ingestion was not meant as self-harm but was rather an attempt to treat alcohol withdrawal with hand sanitizer. He was discharged on hospital day 14. When contacted by phone 2 months later, he reported his vision was unchanged.

## Analysis

The second dialysis treatment (HD#2), which was preceded by a measurable methanol level, allows for an estimation of the efficiency of the Tablo system in treating methanol poisoning. Such assessment requires the use of previously published data, including the data compiled in the 2015 recommendations published by the EXtracorporeal TReatments In Poisoning (EXTRIP) workgroup [4].

First, in the setting of alcohol dehydrogenase (ADH) inhibition (in this case by fomepizole), the endogenous clearance of methanol is quite low relative to HD, with a half-life that averages 52–54 h [5, 6], but is typically even higher in the presence of very high methanol levels such as in this case [5]. This range of half-lives corresponds to reported ranges of renal clearance of 5–6 mL/min and non-renal (presumed pulmonary) clearance of 7–13 mL/min [4]. Methanol has a volume of distribution of 0.6–0.8 L/kg [4, 7] and, given its unicompartmental kinetics, there is no rebound of methanol after HD [8, 9]. In contrast to methanol, the renal clearance of formate, though variable and pH-dependent, is much higher, consistently reported at > 170 mL/min, and the additional clearance of formate by IHD appears small (and of unclear clinical relevance) relative to endogenous clearance [4, 5, 8–11].

In this case, if we conservatively assume an endogenous methanol half-life of 52 h, the serum level of methanol of 64.9 mmol/L (208 mg/dL) at approximately 8 am on day 2 would have decayed to approximately 62.4 mmol/L (200 mg/dL) by the start of dialysis 3 h later. Notably, since methanol does not exhibit rebound after dialysis, the level of 7.2 mmol/L (23 mg/dL) obtained before the end of HD#2 is presumed spurious, likely because it was obtained off the dialysis catheter during treatment, and it is excluded from our analysis.

### Calculation of clearances

We undertook a regression analysis of the methanol levels in logarithmic space, incorporating the previously existing data for the non-dialytic clearance and volume of distribution of methanol into a formal statistical analysis that enabled us to estimate dialytic clearance using the measurements available to us. The unicompartmental kinetics of methanol and the lack of rebound imply a continuous decline of the levels of the toxin over time. Therefore, a measurement obtained at time *t*, *C*(*t*), can be related to the level, *C*(0), obtained at an arbitrary initial time (*t* = 0) according to the logarithmic formula:

$$ \log C(t)=\log C(0)-\frac{K_{\mathrm{ND}}\bullet t}{V}-\frac{K_{\mathrm{Tablo}}\bullet \Delta  {t}_{\mathrm{Tablo}}}{V}-\frac{K_{\mathrm{HD}}\bullet \Delta  {t}_{\mathrm{HD}}}{V} $$

where *K*_ND_ is the non-dialytic (renal + non-renal) clearance set at 16 mL/min (median of data range reported by EXTRIP [4]), *V* is the volume of distribution of methanol (conservatively set to 60 kg for this estimate), ∆*t*_Tablo_ is the total treatment duration with Tablo to time *t*, and ∆*t*_HD_ is the total treatment duration with conventional IHD to time *t.* We set time zero to the time the 64.9 mmol/L (208 mg/dL) level was obtained. Using this formal approach, the estimates for the two clearances were 341 mL/min [95% confidence interval (CI) 252–429 mL/min] for conventional IHD (*K*_HD_) and 239 mL/min (95% CI 173–305 mL/min) for Tablo (*K*_Tablo_). Both of these clearances are well within the range of clearances achieved with IHD previously reported in the literature of 77–400 mL/min (mean 208 mL/min) [4].

Another way to assess the efficacy of methanol clearance in this case is to compare the duration of dialysis required in this case to two previously published methods [8, 12] for estimating the duration of IHD needed to treat methanol poisoning. The total observed treatment time for our patient was 9.42 h, including 305 min (*Δt*_Tablo_) with Tablo during HD#2 and 260 min (*Δt*_HD_) during HD#3 with conventional IHD (accounting for the interruption in HD#3).

The first method, published by Hirsch et al. in 2001 [8], estimates the duration (*t*, in hours) to achieve a methanol level of 5 mmol/L (16 mg/dL) to be:

$$ t=-V\bullet \ln \left(\frac{5}{A}\right)/\left(0.06\bullet k\right) $$

where *V* is the estimate of total body water, *A* is the initial methanol concentration (in mmol/L), and *k* is 80% of the manufacturer-specified dialyzer urea clearance (in mL/min) at the given *Qd*. However, in this specific case, rather than estimating *k* from the dialyzer characteristics, we can use the estimates generated from our in vivo data for methanol clearance specific to both Tablo and conventional IHD (*K*_Tablo_ and *K*_HD_, respectively). Using these two estimated values for methanol clearance as *k* in the Hirsch equation gives an estimated duration of 10.6 and 7.4 h, for Tablo and conventional IHD, respectively, to bring the methanol level down from 62.4 mmol/L (200 mg/dL, the estimated level at the beginning of HD#2) to 5 mmol/L (16 mg/dL), assuming we had used only Tablo or only conventional IHD for both treatments. Since we used Tablo followed by conventional IHD, the total clearance can be estimated as the time-weighted average of the clearances of the two methods:

$$ {K}_{\mathrm{ave}}=\frac{\Delta  {t}_{\mathrm{Tablo}}}{\Delta  {t}_{\mathrm{Tablo}}+\Delta  {t}_{\mathrm{HD}}}\bullet {K}_{\mathrm{Tablo}}+\frac{\Delta  {t}_{\mathrm{HD}}}{\Delta  {t}_{\mathrm{Tablo}}+\Delta  {t}_{\mathrm{HD}}}\bullet {K}_{\mathrm{HD}} $$

which yields a *K*_ave_ of 286 mL/min. Using this *K*_ave_ for *k* in the Hirsch equation yields an estimated treatment time of 8.81 h, which is similar to the observed 9.42 h it took in this case.

Lachance et al. in 2015 published gender-specific equations for predicting treatment duration for high-efficiency HD [12]. Specifically, they proposed the equations of:

$$ t=3.390\bullet \left(\ln \left(\frac{MCi}{4}\right)\right) $$

for women, and:

$$ t=3.534\bullet \left(\ln \left(\frac{MCi}{4}\right)\right) $$

for men, where *MCi* is the initial methanol concentration (in mmol/L) and *t* is the time (in hours) needed to achieve a methanol level of 6 mmol/L (19.2 mg/dL). For our man with an initial methanol level of 64.9 mmol/L (208 mg/dL), one would predict a treatment time of 9.7 h, which closely matches our observed treatment time of 9.42 h using the combination of Tablo and conventional IHD.

## Discussion

Several brands of imported hand sanitizer distributed in the southwest US during the COVID-19 pandemic were found by the US Food and Drug Administration (FDA) in June of 2020 to be contaminated with methanol. This ultimately led to an outbreak of methanol poisoning which resulted in 15 known hospitalizations and at least four deaths in New Mexico and Arizona in May and June of 2020 [2, 13]. Of these 15 patients, nine, including our subject treated with the Tablo device, ultimately required renal replacement therapy (RRT) [2].

As the primary mediator of toxicity is not methanol but formate, the outcome of methanol poisoning depends on more than the presenting methanol level. However, levels greater than 15.6–31.2 mmol/L (50–100 mg/dL) have been associated with an increased risk of death or permanent disability [4, 14]. We did not have access to formate levels in this case, but the initial methanol level of > 156 mmol/L (> 500 mg/dL) is on the same order of magnitude as some of the highest levels recorded in survivors of methanol poisoning [15] and was the highest initial level documented in this particular outbreak tracked by the US Centers for Disease Control [2]. The significant visual impairment noted in this case, though unfortunate, is consistent with previously reported cases of methanol intoxication at levels well below 156 mmol/L (500 mg/dL) that resulted in permanent visual impairment [2, 9, 16, 17].

## Mini-review

### Features of the Tablo Hemodialysis System

Tablo is a next-generation, self-contained hemodialysis system capable of adaptive kidney replacement therapy from hospital to home. Tablo was approved by the FDA for use in hospitals and dialysis centers in 2016 and for in-home use in 2020. Tablo contains three primary components: (1) the Tablo Console, a compact, easily transportable console with integrated water purification, on-demand dialysate production, and touchscreen interface; (2) the Tablo Cartridge, a disposable, single-use organizer with pre-strung bloodlines compatible with any commercially available dialyzer that easily clicks into place; and (3) the TabloHub, a web-based portal enabling Tablo to stay connected with two-way wireless communication, cloud-based system monitoring, treatment analytics, and clinical recordkeeping that can be integrated with electronic medical records. See Table 3 for further device and operating specifications.

**Table 3:** Device and operating specifications for Tablo Hemodialysis System

Approximate device dimensions (cm)	50 (*l*) × 50 (*w*) × 90 (*h*)
*Qb*	Up to 400 mL/min
Extracorporeal circuit volume	140 mL (excluding dialyzer)
Maximum UF rate	2000 mL/h
UF accuracy	±50 mL/h
*Qd*	50–300 mL/min
Dialysate preparation	Standard 45X proportioning
Dialysate concentrates	Bicarbonate or acetate/citrate acid
Dialysis fluid potassium (mEq/L)	0, 1, 2, 3, 4
Dialysis fluid calcium (mEq/L)	0, 1.0, 1.5, 1.75, 2.0, 2.25, 2.5, 2.75. 3.0, 3.25, 3.5
Dialysis fluid sodium (mEq/L)	130–145
Dialysis fluid total buffer (mEq/L)	30–40
Dialysis fluid temperature (C)	35–38
Treatment duration	5 min to 24 h
Prime time	8 min
Incoming water temperature (C)	5–32
Incoming water pressure (PSIG)	30–80
Treatment and maintenance features	UF only
	Programmed sequential therapy (either HD then UF or UF then HD)
	Saline flush, manual or scheduled, with automatic fluid removal adjustment
	Automatic saline bolus with volume tracking
	Integrated blood pressure cuff
	Automated heat disinfection (daily) and chemical disinfection
	Individually replaceable sediment filter, carbon filter, and ultrafilters

*h*, height; *HD*, hemodialysis; *l*, length; *PSIG*, pounds per square inch gauge; *Qb*, blood flow rate; *Qd*, dialysate flow rate; *UF*, ultrafiltration; *w*, width. Source: www.outsetmedical.com

The integrated water purification system includes sediment filter, carbon filter, reverse osmosis system, and ultrafilter, allowing the use of any source of drinking water (as defined by US Environmental Protection Agency standards) to generate dialysate that meets the standards of the Association for the Advancement of Medical Instrumentation. The logistical advantage of being able to use any potable water source and the small device footprint result in increased portability, ease of use, and simplified sterilization, which led to our institutional adoption of Tablo as our default portable IHD device during our initial COVID-19 surge.

In addition, through sensor and software-based innovation, Tablo has been designed with the flexibility to perform dialysis for up to 24 h, allowing it to serve as a single device replacement for both traditional IHD and continuous renal replacement therapy (CRRT) devices or as a cost-effective solution for prolonged intermittent renal replacement therapy (PIRRT, also known as sustained low-efficiency dialysis or SLED) [18]. PIRRT is a hybrid therapy that utilizes low *Qb*, similar to CRRT, with *Qd* and ultrafiltration rates that are intermediate to traditional CRRT and traditional IHD with a typical duration of therapy between 6 and 12 h. PIRRT can be used as either a replacement for CRRT or as a bridge between CRRT and IHD therapies. PIRRT has been shown to have clinically equivalent results to CRRT in the ICU [19, 20]. The use of Tablo, specifically, as a PIRRT device has been shown to be substantially less expensive than similar duration therapy with traditional IHD and CRRT devices [18]. This flexibility has led some hospitals in the US to adopt Tablo as their only RRT device, using it to deliver IHD to hemodynamically stable patients and extended therapies to unstable patients in the ICU. This case report is particularly informative to these institutions as our analysis suggests that the standard of care for the dialytic treatment of toxic alcohol poisoning can be readily achieved with IHD using Tablo.

### The treatment of methanol poisoning

With severe methanol intoxication, early initiation of therapy is critical. Empiric treatment with fomepizole at the outside ED likely had a significant benefit in this case and highlights the importance of initiating treatment prior to the availability of toxic alcohol levels when clinical suspicion is high. This is especially true in most facilities, such as ours, in which toxic alcohol panels take several hours or longer to result. In addition to immediate treatment with an ADH inhibitor such as fomepizole or ethanol, cofactor support (with IV folate or folinic acid for methanol poisoning and/or IV pyridoxine and thiamine for ethylene glycol poisoning) is also recommended in the setting of suspected toxic alcohol poisoning to help shift the metabolism of the parent alcohols away from the toxic acids (e.g., formic acid and oxalic acid) towards less toxic metabolites [4, 14].

Though experts and guidelines recommend somewhat differing thresholds for RRT initiation, prompt RRT is the cornerstone of therapy for severe toxic alcohol poisoning [4, 14, 21, 22]. EXTRIP, for example, recommends RRT for methanol poisoning in all cases with neurologic impairment (seizures, coma, or visual deficit); for metabolic acidosis that is severe (pH ≤ 7.15 or anion gap > 24 mmol/L) or persistent despite supportive care; if methanol levels are greater than 15.6 to 21.8 mmol/L (50–70 mg/dL), depending on the concomitant use of fomepizole or ethanol; or in the context of impaired kidney function [4]. In cases in which the need for RRT is unclear or borderline, consultation with toxicology is vital. Whenever IHD is employed, strong consideration should be given to the use of a higher *K*_*O*_*A* dialyzer and/or a longer duration of treatment than that of a traditional IHD session in order to maximize methanol clearance. While the Hirsch [8] and Lachance [12] equations can be used to predict the duration of IHD required based on initial methanol levels, the initial treatment duration, as is in this case, is often selected empirically, though some have recommended a longer initial empiric duration of 8 h of IHD [4, 9]. Regardless, serial methanol levels should be monitored if available, and RRT should be continued until both the pH and anion gap have normalized and the methanol level is less than 6.2 mmol/L (20 mg/dL) [4]. Importantly, fomepizole is dialyzable and therefore requires dose adjustment if being used in the context of RRT. Nephrologists should also be aware that (though in-line heparin was used in this case) the most recent EXTRIP guideline recommends avoiding systemic anticoagulation with RRT due to reports of intracranial hemorrhage occurring in a substantial minority of patients with methanol poisoning [4, 23].

The average reported clearances of methanol with IHD, CRRT, and peritoneal dialysis are 208, 37, and 37 mL/min, respectively, underlying the superiority of IHD, whenever feasible, for treating methanol poisoning [4]. In this case, the achieved clearance well above the reported mean for IHD with both Tablo, at a *Qd* of 300 mL/min, and a traditional IHD machine, at a greater than two times higher *Qd*, underscores the modest impact of *Qd* on methanol clearance.

### Impact of dialysate flow rate on small molecule clearance

As described in the original Michaels’ equation [24], clearance (*K*) of urea (and that of other small dialyzable solutes) in countercurrent HD is a function of *Qd*, *Qb*, and the product of the mass transfer coefficient and membrane surface area (*K*_*O*_*A*) of a given dialyzer:

$$ K= Qb\bullet \left(\frac{\exp \left(\frac{KoA\left(1-\frac{Qb}{Qd}\right)}{Qb}\right)-1}{\exp \left(\frac{KoA\left(1-\frac{Qb}{Qd}\right)}{Qb}\right)-\frac{Qb}{Qd}}\right) $$

Within the ranges of *Qb*, *Qd*, and *K*_*O*_*A* that are typically used for IHD, the mathematical impact of *Qd* is the least of these three variables. While IHD is often prescribed with a *Qd* set to 1.5 to 2 times *Qb*, multiple recent studies, including both empiric human data [25–29] and analysis of modeled data, suggest the effect on *Kt*/*V* of decreasing *Qd* to 300–400 mL/min is modest. Of note, this has been specifically demonstrated with the Revaclear dialyzer, which, like other modern dialyzers, has been designed with enhanced dialysate flow distribution which minimizes the effect of *Qd* on *K*_*O*_*A* [28, 29].

The maximum *Qd* accommodated by Tablo is 300 mL/min. A recent kinetic modeling analysis of the effect of decreasing *Qd* from 500 to 300 mL/min at *Qb* rates of 300–400 mL/min concluded that the resulting drop in equilibrated *Kt/V*_urea_ would be relatively small at 0.12–0.22, a difference that could be nearly fully counteracted by using a dialyzer with a higher *K*_*O*_*A* (1480 versus 1170 mL/min) and extending treatment time by 15 min [30]. A recent in vivo study of six patients on maintenance dialysis sequentially treated with Tablo with a *Qd* of 300 mL/min and a conventional IHD machine (Gambro Phoenix) with *Qd* of 500 mL/min yielded clearance curves for urea, potassium, phosphate, and β_2_-microglobuin which were remarkably similar, with no statistically significant difference in the levels of any of the four solutes by the end of 4 h of treatment despite the differing *Qd* rates [27].

## Conclusion

This is the first reported case of the use of the Tablo Hemodialysis System for the treatment of toxic alcohol poisoning. Our analysis of this case illustrates that IHD with Tablo can achieve a clearance of methanol that is well within the previously published standard of care. This clearance could likely be enhanced further with a high *K*_*O*_*A* dialyzer. Hemodialysis, with any device, remains an effective way to treat toxic alcohol poisoning, especially when combined with pharmacologic ADH antagonism and when optimized with extended treatment times and use of a higher *K*_*O*_*A* dialyzer.

## Data Availability

All data are either included or available upon request.

## Abbreviations

ADH: Alcohol dehydrogenase
CI: Confidence interval
CRRT: Continuous renal replacement therapy
CT: Computed tomography
COVID-19: Coronavirus disease 2019
ED: Emergency department
EMS: Emergency medical services
EXTRIP: EXtracorporeal TReatments In Poisoning workgroup
FDA: United States Food and Drug Administration
HD: Hemodialysis
IHD: Intermittent hemodialysis
*K*: Clearance
*K*_*O*_*A*: Product of the mass transfer coefficient and membrane surface area of a dialyzer
PIRRT: Prolonged intermittent renal replacement therapy
*Qb*: Blood flow rate
*Qd*: Dialysate flow rate
SLED: Sustained low-efficiency dialysis
US: United States
